# Analysis of chemotypes and their markers in leaves of core collections of *Eucommia ulmoides* using metabolomics

**DOI:** 10.3389/fpls.2022.1029907

**Published:** 2023-01-09

**Authors:** Yide Meng, Qingxin Du, Hongyan Du, Qi Wang, Lu Wang, Lanying Du, Panfeng Liu

**Affiliations:** ^1^ Research Institute of Non-Timber Forestry, Chinese Academy of Forestry, Zhengzhou, China; ^2^ Key Laboratory of Non-timber Forest Germplasm Enhancement & Utilization of National Forestry and Grassland Administration, Zhengzhou, China

**Keywords:** self-organizing map, random forest, non-targeted metabolomics, chemotype classification, marker screening

## Abstract

The leaves of *Eucommia ulmoides* contain various active compunds and nutritional components, and have successively been included as raw materials in the *Chinese Pharmacopoeia*, the *Health Food Raw Material Catalogue*, and the *Feed Raw Material Catalogue*. Core collections of *E. ulmoides* had been constructed from the conserved germplasm resources basing on molecular markers and morphological traits, however, the metabolite diversity and variation in this core population were little understood. Metabolite profiles of *E. ulmoides* leaves of 193 core collections were comprehensively characterized by GC-MS and LC-MS/MS based non-targeted metabolomics in present study. Totally 1,100 metabolites were identified and that belonged to 18 categories, and contained 120 active ingredients for traditional Chinese medicine (TCM) and 85 disease-resistant metabolites. Four leaf chemotypes of the core collections were established by integrated uses of unsupervised self-organizing map (SOM), supervised orthogonal partial least squares discriminant analysis (OPLS-DA) and random forest (RF) statistical methods, 30, 23, 43, and 23 chemomarkers were screened corresponding to the four chemotypes, respectively. The morphological markers for the chemotypes were obtained by weighted gene co-expression network analysis (WGCNA) between the chenomarkers and the morphological traits, with leaf length (LL), chlorophyll reference value (CRV), leaf dentate height (LDH), and leaf thickness (LT) corresponding to chemotypes I, II, III, and IV, respectively. Contents of quercetin-3-O-pentosidine, isoquercitrin were closely correlated to LL, leaf area (LA), and leaf perimeter (LP), suggesting the quercetin derivatives might influence the growth and development of *E. ulmoides* leaf shape.

## 1 Introduction

Although have not yet been fully explored, plant metabolites are important sources for food, medicines, health care products, feed additives and some industrial materials. Plants were estimated to produce above 200,000 metabolites in nature (Dixon, 2003), while a single species was thought to contain 4,000~20,000 metabolites that exerted a wide range of effects on growth, development, and interactions with biotic or abiotic environments (Fernie et al., 2004; Wang et al., 2019a). *E. ulmoides* had been widespread in continents of the northern hemisphere according to the fossil records, however, after the quaternary glaciation it only survived in northern and central China (Call and Dilcher, 1997). By the introduction and cultivation, at present *E. ulmoides* is widely planted in China as one of the important tree species of national strategic resources, which horizontally distributed in 24.5 ~ 41.5° N, 76 ~ 126° E, and vertically below 2500 m (Li et al., 2019). Many valuable metabolites including iridoids, flavonoids, phenylpropanoids, and lignans had been extracted from *E. ulmoides* leaves, and those exerted good functions in antibacterial, anti-inflammatory, anti-oxidant, immune regulation, and hypoglycemic, and anti-hypertension (Zhu et al., 2018; Wang et al., 2019b). *E. ulmoides* leaves were listed as traditional medicinal herbs in the 2005 and 2015 editions of the Chinese Pharmacopoeia, and included in the pilot project list for drug & food homologation by the National Health Commission in 2019 (Gong et al., 2021). Besides, the extracts of *E. ulmoides* leaves were stipulated for uses of growing-finishing pigs, fish, and shrimp in the Catalogue of Feed Additives (2013) that issued by the Ministry of Agriculture of China (Yang et al., 2021).

Phytochemotype was regarded as a plant phenotype differentiated by content, composition or structure of the endogenous chemicals within a species, which indicated the intraspecific variation and diversity (Rovesti, 1957; Eilers et al., 2021). Analyses of phytochemotype classification and its formation mechanism had both theoretical and practical values in guiding the development & utilization, quality evaluation and oriented cultivation of plant germplasm resources (Desjardins, 2008). Although standards of chemotype classification and nomenclature for different plant species have not been unified (Hua et al., 2009), intraspecific chemotype variations were found and studied in several tens plants including *Agastache rugosa, Cannabis sativa*, and *Valeriana jatamansi* since the concept proposed (He et al., 2018; Jin et al., 2021; Dang et al., 2022). Because of the development of statistical analysis for high dimensional data, and the maturity of chromatograph-mass spectrometer technologies, the high-throughput detection of plant metabolomics provided whole and new perspectives for accurate chemotype classification and evaluation, and related metabolic regulatory mechanisms (Deng et al., 2020; Yang et al., 2020). Metabolites in different plant chemotypes could be the causes or markers of the morphological traits (Saito, 2013). Co-expression analysis between metabolites and morphological traits facilitated early selection and prediction of complex traits in breeding, and that had been successfully applied to several staple crops (Shepherd et al., 2006; Wei et al., 2017; Shi et al., 2020). *E. ulmoides* was a monotypic species of Eucommiaceae. Germplasm genetic diversity of *E. ulmoides* had been evaluated by morphological traits, major active metabolites, and a number of molecular markers from RAPD to SNP (Wu et al., 2011; Yao et al., 2012; Yu et al., 2015; Du et al., 2017; Liu et al., 2022). Most results tended to believe that the genetic diversity of *E. ulmoides* at the intra-population level was higher than at the inter-population level, and its genetic distance was not evidently correlated to geographical distribution. However, at systematic chemotypic level, *E. ulmoides* germplasm resources were not been fully evaluated and classified except of the male flower core collections (Liu et al., 2020). *E. ulmoides* core collections from nearly 2,000 germplasm resources had been constructed and conserved in the National Germplasm Resources Bank of Major Famous Tree Species in the North (34°55′22″ N, 113°46′16″ E), Yuanyang, Henan Province (Li et al., 2018a).

In this study, we conducted a large scale metabolomics study by GC-MS and LC-MS/MS in leaves of 3 clonal replicates of 193 core collections (579 individuals) form 43 geographic origins that grew under similar growth environment and cultivation practices, to determine the possible leaf chemotypes and chemical markers across the collections. We processed the non-targeted metabolomics data by two clustering methods, K-means and SOM, and evaluated the results by comprehensive uses of RF model, OPLS-DA, and targeted determination of 13 metabolites. Additionally, we analyzed the correlations between the leaf metabolites and morphological traits, and the morphological markers of the chemotypes. These results would provide valuable references and be conductive to the germplasm collection strategy, directed breeding, quality control, and leaf resources utilization of *E. ulmoides*.

## 2 Materials and methods

### 2.1 Plant materials and sampling

Six individuals of 193 core collections have been planted after grafting propagation at tree spacing of 3 m × 3 m and implemented by basically consistent cultivation measures and managements in the *E. ulmoides* germplasm pool since 2013. Mature leaves grew at the middle annual branch were sampled from 2~4 individuals of each core collection in late August 2020. The samples were separated to 6 biological replications for GC-MS non-targeted determination, and to 3 biological replications for LC-MS/MS non-targeted determination, respectively. After sampling the materials were firstly frozen in liquid nitrogen, then stored at -80°C in laboratory. Samples for HPLC targeted determination were sampled in late August 2021 and followed the similar methods. Geographical origins of 193 collections and their leaf morphology characters were summarized and analyzed in **Supplementary Table 1**.

### 2.2 GC-MS analysis

Powdery sample was weighed (ca. 80.0 mg) after ground by liquid nitrogen and was added with 60 µL 0.2 mg·mL^-1^ ribitol and 1.5 mL extraction solvent (methanol, chloroform and water in volume ratio of 2.5:1:1) for 30 min sonication in cold water bath. After centrifuging the mixture at 12,000 rpm for 10 min at 4°C, the supernatant of 1ml was transferred, followed by the addition of 400 µL water. The supernatant of 100 µL was transferred and dried by nitrogen after the mixture vortexed for 30 s and centrifuged at 12,000 rpm for 5 min at 4°C. The methoxyamine hydrochloride of 60 µL (20 mg·mL-1) was added in pyridine to the resident solution and shaken for 90 min at 37°C for derivatization. Then 100 µL MSTFA and 1% TMCS were added to the mixture and shaken 30 min at 37°C. At last, the solution was derived at room temperature for 30 min and transferred to the liner tube for test after filtered by 0.50 µm filter membrane (Weckwerth et al., 2004).

The injection of 1 µL of solution into a Thermo scientific Trace 1310 GC-MS system (Thermo Fisher Scientific, USA) was performed in the non-split mode. Separation was carried out on a non-polar DB-5 capillary column (30 m × 0.25 mm I.D., J&W Scientific, USA), with high purity helium as the carrier gas at a constant flow rate of 1.0 mL/min. The column temperature program was of 80°C for 5 min, following ramped at 10°C. min to 195°C and held for 4 min, and then ramped at 3°C. min to 260°C and held for 6 min, and finally ramped at 4°C. min to 305°C and held for 5 min. Temperature of the ion source, the transmission line and the inlet temperature was set to 310°C, 260°C, and 280°C, respectively. Full scan mode (SCAN) was employed and the mass scan range was of 50~600 m/z. The quality control (QC) sample was prepared by mixing aliquots of the samples to be a pooled sample. To monitor and assess GC-MS system stability, 1 injection of QC sample was tested behind every 10 injections of the normal samples (Subbaraj et al., 2019).

### 2.3 LC-MS/MS analysis

The powder sample of 100 mg was ground and weighed after vacuum freeze-drying The sample was added with 1 mL of 50% methanol and shaken for 1 h. Then the mixture was centrifuged at 14,000 rpm for 10 min after placed in a refrigerator at 4°C overnight. The supernatant was transferred to an inner tube for test after filtered through 0.22 µm membrane.

The injection of 5 µL of solution into liquid chromatography Q-Exactive Orbitrap mass spectrometry (Thermo Fisher Scientific, USA) was performed by the autosampler at 10°C. Chromatographic separation was carried out on the Hypersil GOLD™ AQC18 column (2.1 mm × 100 mm, particle size 1.9 µm) at 30°C applying the following binary gradient at a flow rate of 300 µL. min^-1^: 0-2.5 min, isocratic 90%-95% A (water/formic acid, 99.9/0.1 [v/v]), 5%-10% B (acetonitrile/formic acid, 99.9/0.1 [v/v]); 2.5-6 min, 10%-12% B; 6-10 min, 12%-15% B; 10-13 min, 15%-17% B; 13-14 min, 17%-25% B; 14-20 min, 25%-80% B; 20-22 min, 80%-100% B 22-25 min, 100% B; 25-25.1 min, linear from 100% to 5% B; 25.1-28 min, 5% B. Eluted compounds were detected from m/z 70 to 1,050 using a Q-Exactive Orbitrap mass spectrometer equipped with an HESI electrospray ion source in positive and negative ion modes using the following instrument settings: spray voltage, ± 3.2 kV; Full MS resolution, 70,000; dd-MS^2^ resolution,17,500; collision gas, high purity N_2_; step collision energy, 20/40/60 V. QC samples were scanned at interval of 10 samples (Li et al., 2018b).

### 2.4 HPLC analysis

Powder samples of 500 mg were ground and weighed after vacuum freeze-drying. The sample was added with 10 mL of 60% methanol and extracted by ultrasonication for 30 min. Then the mixture was centrifuged at 12,000 rpm for 10 min after placed in a refrigerator at 4°C overnight. The supernatant was transferred to injection bottle for test after filtered through 0.22 µm membrane.

The injection of 5 µL solution into high performance liquid chromatography (Waters ACQUITY Arc system, USA) coupled with a 2998 photodiode array (PDA) detector was performed. Chromatographic separation was carried out on Thermo Syncronis C18 column (250 mm × 4.6 mm, particle size 5 µm) at 30°C applying the following binary gradient at a flow rate of 800 µL·min^-1^: 0-10 min, isocratic 83%-95% A (water/formic acid, 99.8/0.2 [v/v]), 5%-17% B (acetonitrile/formic acid, 99.9/0.1 [v/v]); 10-25 min, 17%-20% B; 25-30 min, 20%-30% B; 30-50 min, 30%-50% B; 50-55 min, 50%-70% B; 55-57 min, linear from 70% to 5% B. The detection wavelengths were set to 204 nm for aucubin, 239 nm for geniposide acid and asperuloside, 247 nm for protocatechuic acid, 276 nm for catechin and pinoresinol diglucoside, 264 nm for methyl gallate, genipin and rutin, 325 nm for chlorogenic acid, and 364nm for isoquercitrin, quercetin and kaempferol. Above 13 compounds were identified respectively by comparison of the retention time to corresponding standard, and their contents were determined according to the external standard method (**Supplementary Table 2** and **Supplementary Figure 1**).

Standards of aucubin, chlorogenic acid, rutin, methyl gallate, and kaempferol with purity≧98% were purchased from Shanghai Anpu Experimental Technology Co., Ltd. standards of genipin, catechin, and asperuloside with purity = 98% were from Shanghai Yuanye Bio-technology Co., Ltd. and the standards with purity = 98% including geniposide acid, protocatechuic acid, quercetin, and pinoresinol diglucoside were from Chengdu Manst Bio-technology Co., Ltd. Isoquercitrin standard with purity = 97% was purchased from National Institute for the Control of Pharmaceutical and Biological Products (Beijing, China).

### 2.5 Identification and quantification

The MS spectra data determined by GC-MS and LC-MS/MS were firstly converted to analysis base file (Abf) format by ABF Converter. Initial characteristic features were obtained after peak detection, peak identification, deconvolution, peak alignment, filtering, characterization and retention time correction of MS spectra data by MS-DIAL (V4.38) (Tsugawa et al., 2015). The main MS-DIAL parameter settings are provided in **Supplementary Table 3**. The feature values were removed when the features were undetected in 80% of the biological samples, then the extrapolated values were filled using the k-nearest neighbor algorithm (KNN) (Dunn et al., 2011).

High quality features in QC samples were obtained after their peak values calibrated comparatively by three algorithms including Random forest (RF), locally weighted scatter plot smoothing (LOESS), and support vector regression (SVR). For GC-MS features, the criterion of optimal algorithm was relative standard deviation (RSD) of the peak values of the reserved features below 50% and their quantity proportion in total features above 70%, and for LC-MS/MS features, the criterion was RSD below 30% and the proportion above 70%. RF and LOESS analyses were completed by statTarget R package (V1.16.1), and SVR analysis was completed by MetNormalizer R package (V1.0) (Luan et al., 2018; Shen et al., 2019).

The metabolites determined by GC-MS were annotated basing on the Fiehn database (https://fiehnlab.ucdavis.edu/) and retention index (retention index, RI) with C_8_~C_40_ n-alkanes (Lai et al., 2018), and those determined by LC-MS/MS were annotated basing on Massbank (http://www.massbank.jp), GNPS (http://gnps.ucsd.edu) and ReSpect (http://spectra.psc.riken.jp/) (Tsugawa et al., 2015). Traditional Chinese Medicine Systems Pharmacology Database and Analysis Platform (TCMSP) (https://tcmsp-e.com/tcmsp.php) was also used to screen the active ingredients for traditional Chinese medicine (TCM) and disease-resistant metabolites in the annotated metabolites (Li et al., 2022). For large-scale samples, unstable injection often occurred in the determination of GC-MS, which could result in variable metabolome data in the biological duplicates, PCA analysis were carried out to remove 3 outliers of each collection. The final metabolome data were combined from the GC-MS and LC-MS/MS after removing repeat annotated metabolites.

### 2.6 Statistical analysis

The data set was firstly log standardized, and then averaged to enhance normality. SOM unsupervised clustering and K-means were used to perform the chemotype classification by analyzing the metabolite composition and content, and RF discriminant model was used to evaluate difference in the chemotypes. The optimal clustering method and the number of chemotypes classification were identified by evaluating the classification effect of the two clustering methods. SOM analysis was carried out by Python 3.6 software, K-means and RF analyses were carried out by R 3.6 software, in which the tree number of RF modle was set to 1,000 (Chavent et al., 2021). Principal component analysis (PCA) was also used by SIMCA-P (V14.0) software to evaluate the difference in the germplasms (Saccenti et al., 2014). The differences between certain chemotype and the other chemotypes were evaluated by OPLS-DA, in which the values of R^2^Y(cum) and Q^2^Y(cum) were used to judge the validity of the model (Triba et al., 2015). Latent biomarkers of each chemotype were screened basing on the OPLS-DA results and using 4 criteria: (1) a variable importance of projection (VIP) value ≥2, (2) a Student’s t-test p value < 0.05, (3) a fold change (FC) value > 0.5, and (4) a mean decrease accuracy (MDA) value ≥ 2 in the RF model, and finally the results were shown in volcano plots using R software (V3.6).

Metabolite co-expression network was constructed to explore potential correlations between metabolites and leaf morphological traits using WGCNA by R package (V1.70), and a visual correlation network between key metabolite and leaf morphological traits was construct using Cytoscape software (V3.7.0) (DiLeo et al., 2011). RF regression model using metabolites in the key module as independent variables and leaf morphological traits as dependent variables was developed, in which the predictability was evaluated by determination coefficient R^2^. In addition, leaf morphological trait-related markers were screened according to the variable importance measured in the RF model (Dan et al., 2021). Finally, leaf morphological traits were predicted by least absolute shrinkage and selection operator (LASSO) regression using the non-targeted metabolome data (Xu et al., 2017).

## 3 Results

### 3.1 Data correction and metabolite annotation

TIC plots of typical QC samples of GC-MS and LC-MS/MS untargeted metabolomics assays were shown in **Supplementary Figure 2**. For both GC-MS and LC-MS/MS mass spectra data of *E. ulmoides* leaves, RF model was more optimal and suitable for QC calibration than the LOESS and SVR algorithms according to the employed criterions, and 85.38%, 82.25% and 71.94% of the total features were eserved in QC samples determined by GC-MS, ESI+ and ESI- mode of LC-MS/MS, respectively (**Supplementary Table 4** and **Supplementary Figure 3**). Totally 7,438 initial characteristic features, including 5,910 high quality features, were obtained from GC-MS mass spectra data, and 30,881 and 15,553 initial characteristic features, including 27,526 and 14,633 high quality features, were obtained from LC-MS/MS mass spectra data in the ESI^+^ and ESI^-^ mode, respectively (**Supplementary Table 4**). In addition, RSD of the internal standard ribitol by GC-MS determination was 30.84%, and that also showed the calibrated results were stable and reliable after QC calibration by RF model (**Supplementary Figure 3**).

**Figure 1:**
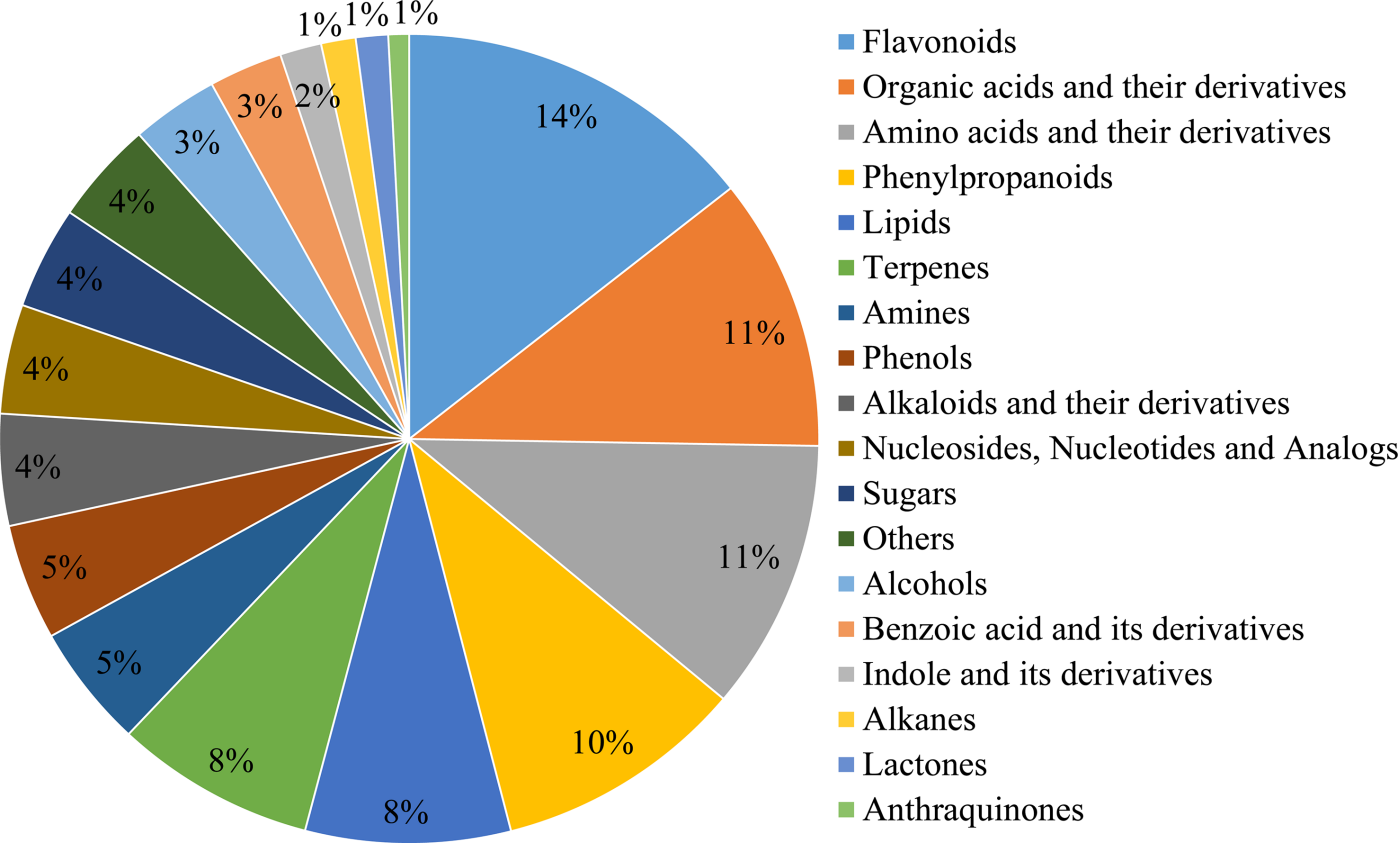
Categories of the annotated metabolites in leaves of *E. ulmoides*.

For metabolite annotation, 209 and 891 metabolites were annotated from the GC-MS and LC-MS/MS determination, respectively, and finally 1,100 metabolites were annotated in leaves of 193 *E. ulmoides* core collections after metabolome assay (**Supplementary Tables 5**, **6**). The metabolites broadly classified into 18 categories according to KEGG database, the top 6 of which were flavonoids, organic acids, amino acids, phenylpropanoids, lipids, and terpenoids, accounting for 14%, 11%, 11%, 10%, 8%, and 8% of the total metabolites, respectively (**Figure 1**). 120 active ingredients were identified among the annotated metabolites through the TCMSP database. In addition, 85 disease-resistant metabolites were identified and functioned to at least one disease (**Supplementary Table 7**), with 10, 30, 19, 43, 23, and 38 metabolites of effects on anti-cancer/tumor, anti-diabetic, anti-cardiovascular, anti-hypertensive, anti-atherosclerotic, and anti-thrombotic, respectively. The above information indicated that *E. ulmoides* leaves were rich in medicinal health substance and could be excellent raw material for pharmatical use.

### 3.2 Chemotype classification

**Figure 2:**
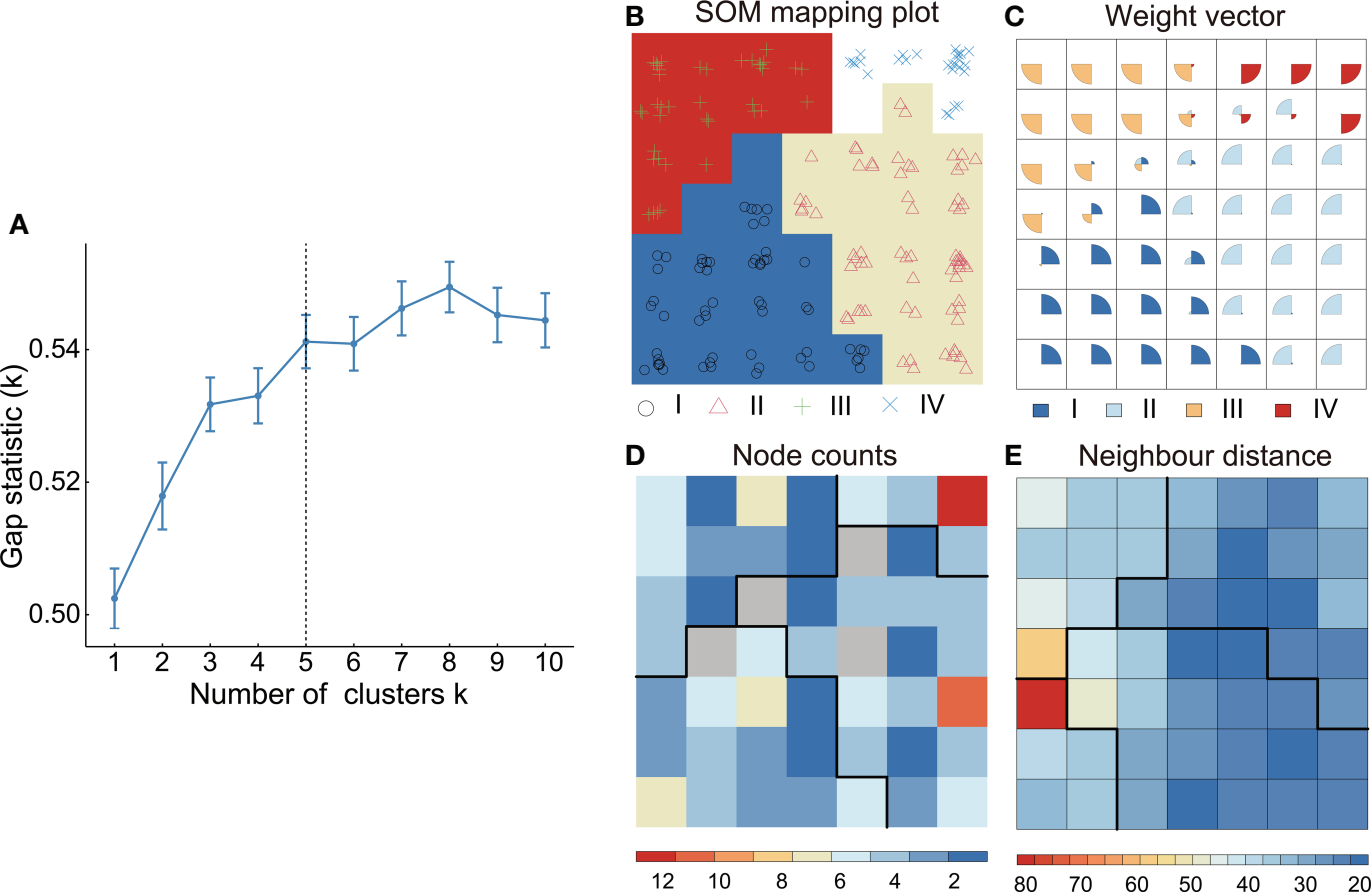
Chemotype classification by K-Means cluster and SOM cluster. **(A)** Number of clusters K *via* the K-means gap statistic. **(B)** Topological mapping plot of the SOM cluster. **(C)** Distribution patterns of the samples and variables of the SOM cluster. **(D)** Variable allocations in each grid node of the SOM cluster. **(E)** Distance of neighboring grid nodes of the SOM cluster.

A matrix consisted of 193 samples ×1,100 metabolites was used to classify the potential chemotypes of *E. ulmoides* leaves. According to K-Means gap statistic, the whole collections were suitable to divide into 4, 5, and 6 groups by K-Means cluster, and incomplete coincidently, similar results were obtained by SOM cluster (**Figure 2A**). To determine which one was the optimal classification method, discriminant RF model was employed to evaluate the results of K-Means and SOM basing on the content of the annotated metabolites. 75% of the 193 core collections were selected as the training set for constructing the prediction model, and the other 25% as the independent set for cross validation. Four chemotypes classified by SOM clustering was considered an optimal result, for the average prediction accuracy of the training set and independent set were highest in the RF model, respectively reaching to 92.52% and 91.30% (**Table 1**).

**Table 1:** Evaluation of the chemotypes classified by SOM and K-means by RF model.

Number of classification	Average prediction accuracy of SOM classification (%)		Average prediction accuracy of K-Means classification (%)	
	Training set	Independent set	Training set	Independent set
4 chemotypes	92.52	91.30	89.04	89.36
5 chemotypes	89.19	86.96	82.35	89.36
6 chemotypes	79.73	84.78	80.27	89.36

Topological mapping maps of SOM showed the projection of the metabolome dataset from high-dimension to low-dimension. For topological mapping maps of four chemotypes classified by SOM, 7 × 7 rectangular topology was selected as the final output layer after 100 iterations of training on the data matrix, in which the minimum quantization error and topology error were taken as training termination criteria. In the obtained topological mapping plots, **Figure 2B** showed that four modules were classified in the samples and similar patterns were expressed in the same group, **Figure 2C** showed the distribution patterns of the samples and variables, with weight vector of the node representing the variables mapped to the node and the fan charts in the grids representing the sample size in the weight vector, **Figure 2D** showed the variable allocations in each grid node, with even allocations in the grid nodes representing accurate classification of the corresponded groups, **Figure 2E** showed the distances between the grid nodes and its adjacent nodes, with the distances lengthening followed to the difference in the nodes increasing. Finally, the 193 collections were classified into four leaf chemotypes, and for each chemotype, consisted of 58, 69, 39, and 27 collections, respectively (**Supplementary Table 8**).

### 3.3 Chemotype comparison

**Figure 3:**
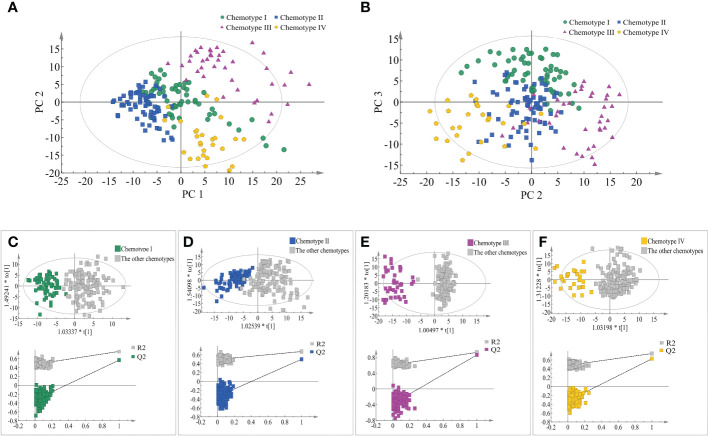
Comparisons of the four classified chemotypes. **(A)** The four chemotypes compared by PC1 and PC2 of PCA model. **(B)** The four chemotypes compared by PC2 and PC3 of PCA model. **(C)** Comparisons between chemotype I and the other chemotypes by OPLS-DA model. **(D)** Comparisons between chemotype II and the other chemotypes by OPLS-DA model. **(E)** Comparisons between chemotype III and the other chemotypes by OPLS-DA model. **(F)** Comparisons between chemotype IV and the other chemotypes by OPLS-DA model.

The separation and similarity across the chemical groups were examined using PCA model, in which 12 principal components were obtained that accounting for 42.6% of the total variance. PC1, PC2, and PC3 accounted for 10.70%, 8.16%, and 5.91% of the total variance, respectively. In the score plot of PC1 and PC2, chemotypes III and chemotype IV were well separated but chemotype I and chemotype II showed partial overlap, while in the score plot of PC1 and PC3 (**Figure 3**), each chemotype was evidently separated. However, no evident correlation was observed between the leaf chemotypes and their geographical origins in the PCA plots. Four supervised OPLS-DA models were constructed to measure the differences between a specific leaf chemotype and the other three chemotypes. From the results of ranking test in each group, values of R^2^Y and Q^2^ of the model after Y replacement were lower than those of the original model as conducting the replacement validation (**Figure 3** and **Supplementary Table 9**), and that indicated the employed OPLS-DA models had good robustness and each classified leaf chemotype was of special characteristic.

### 3.4 Chemomarkers screening

**Figure 4:**
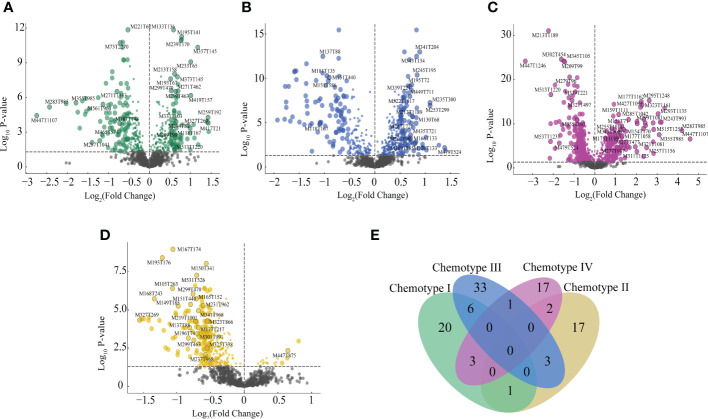
Screened chemomarkers of the four chemotypes. **(A)** Chemomarkers of chemotype (I) **(B)** Chemomarkers of chemotype II. **(C)** Chemomarkers of chemotype III. **(D)** Chemomarkers of chemotype IV. **(E)** Venn plot of the chemomarkers in four chemotypes.

VIP values, P-values, FC values, and MDA values were used to build the criteria of chemomarkers screening. A total of 103 markers were screened in the four chemotypes, in which 30, 23, 43, and 23 markers corresponded to chemotype I, chemotype II, chemotype III, and chemotype IV, respectively. In addition, 20, 17, 33, and 17 markers were found exclusive to the chemotype I, chemotype II, chemotype III, and chemotype IV, respectively, and that suggested these chemomarkers were divergent and could be effective in indicating the chemotypes (**Figure 4** and **Supplementary Table 10**). For chemotype I, the chemomarkers mainly belonged to flavonoids and phenylpropanoids, and three terpenes of 20 content up-regulated chemomarkers were TCM active ingredients. Chemotype II contained the only lactone marker, linderalactone, also identified as active ingredients for TCM and disease-resistant metabolites, and contained the only indole marker, indoline. For chemotype III, the chemomarkers were almost terpenes, lipids, and organic acids, and three of 31 content up-regulated chemomarkers were TCM active ingredients and one up-regulated chemomarkers belonged to disease-resistant metabolites. For chemotype IV, the chemomarkers were mainly phenolics and phenylpropanoids, and contained one up-regulated chemomarkers belonged to the active ingredients for TCM.

RF model was employed to evaluate the accuracy of the chemomarkers in distinguishing the chemotype. Average prediction accuracy of the training set and independent test set for the four chemotypes were 92.52% and 84.78% with AUC value of the independent set 0.972, and specially, the prediction accuracy for chemotype III reached to 100% (**Supplementary Table 11**). Therefore, the results from RF model also showed the chemomarkers were steady to classify the leaf chemotypes of *E. ulmoides*.

### 3.5 Target validation of the chemotypes by 13 metabolites

Contents of 13 important metabolites of *E. ulmoides* leaves frequently followed in previous studies were determined in 193 core collections, as spot checks on the non-targeted results of the chemotype classification and chemomarker screening. Except for three metabolites, methyl gallate, pinoresinol diglucoside, and genipin, 10 metabolites showed similar varied tendency in each chemotype basing on the comparisons between results of the non-targeted and targeted determination (**Supplementary Figure 4**). Specifically, isoquercitrin and kaempferol, chemomarkers of the chemotype I, aucubin and chlorogenic acid, chemomarkers of the chemotype III , their contents in the non-targeted results were down regulated in they indicated chemotypes when comparing to the other types and that was observed exactly consistent with the targeted results by HPLC determination.

### 3.6 Morphological markers of the chemotypes

**Figure 5:**
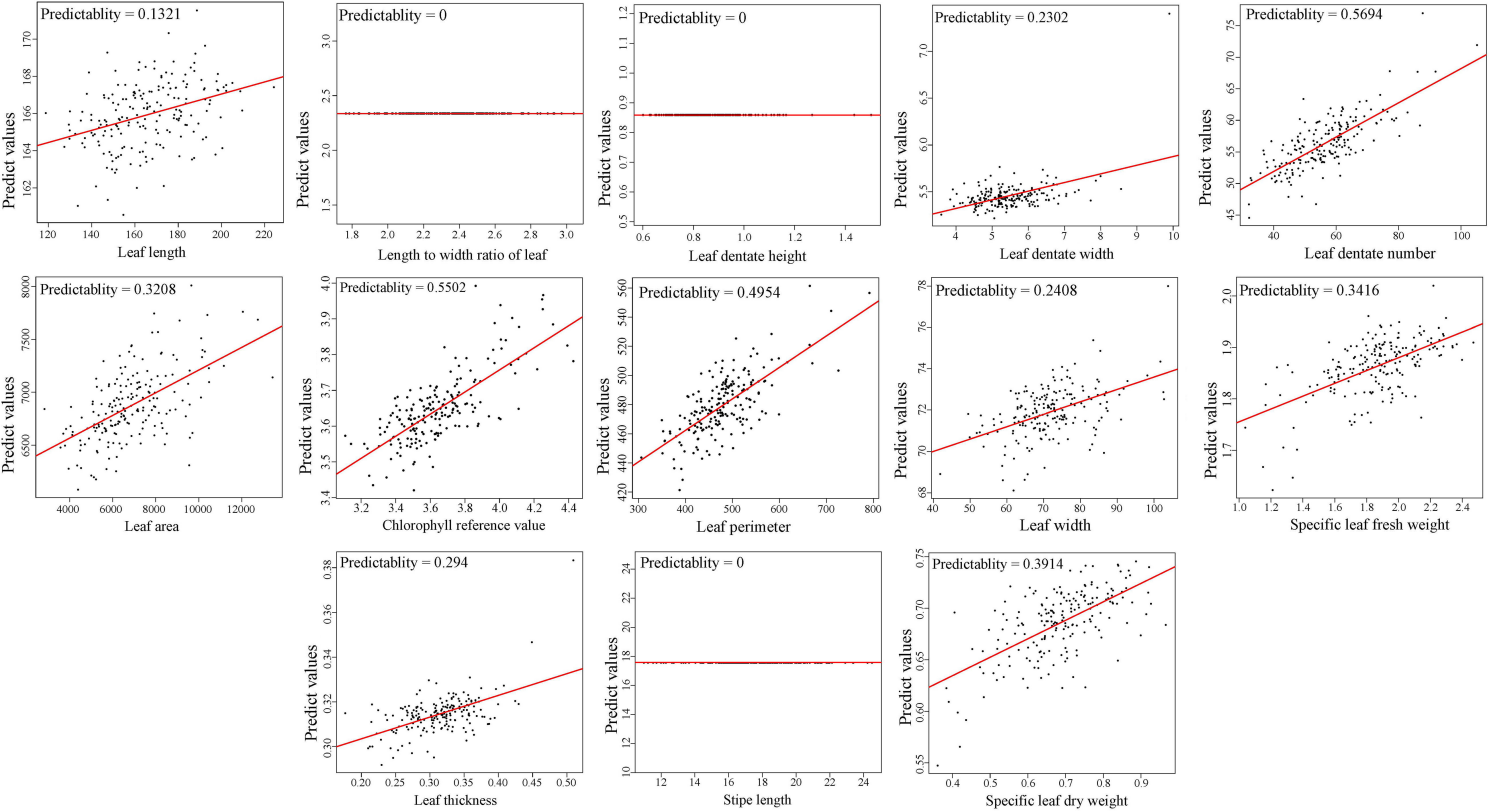
Average predictability of the 13 morphological traits by LASSO model.

LASSO model was used to predict the leaf morphological traits basing on contents of 1,100 metabolites, to assess the correlations between leaf metabolites and morphological traits, and examine the feasibility of screening morphological markers. Average predictability of the 13 morphological traits was 0.3566. The top four predictive values were 0.5694, 0.5502, 0.4954, and 0.3914, respectively, corresponding to leaf dentate number (LDN), chlorophyll reference value (CRV), leaf perimeter (LP), and specific leaf dry weight (SLDW) (**Figure 5**), which indicated a certain degree of associations between the metabolites and morphological traits in *E. ulmoides* leaves. Significant differences were observed by ANOVA in four leaf morphological traits referring to SLFW, CRV, leaf dentate height (LBH), and leaf thickness (LT) among the four leaf chemotypes. The average values of SLFW and CRV were highest in chemotype II, and value of LBH was highest in chemotype III, while value of LT was highest in chemotype IV after multiple comparisons (**Supplementary Table 12**).

**Figure 6:**
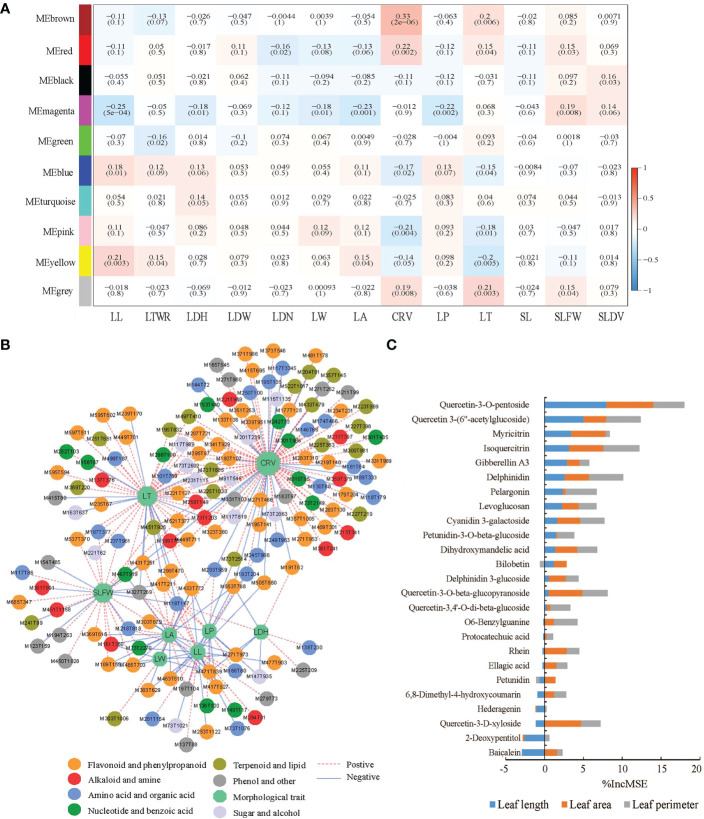
Correlation between the metabolites and the morphological traits of E ulmoides leaves. **(A)** Heat map of the correlations between the modules obtained by WGCNA and the morphological traits. **(B)** Correlation analysis between 377 metabolites in six key metabolite modules and eight morphological traits. **(C)** IncMSE% in the RF regression for 25 metabolites in the Memagenta module. (LL, Leaf length; LTWR, Length to width ratio of leaf; LDH, Leaf dentate height; LDW, Leaf dentate width; LDN, Leaf dentate number; LW, Leaf width; LA, Leaf area; CRV, Chlorophyll reference value; LP, Leaf perimeter; LT, Leaf thickness; SL, Stipe length; SLFW, Specific leaf fresh weight; SLDW, Specific leaf dry weight).

Nine metabolite modules contained 601 metabolites were obtained by WGCNA between 1100 metabolites and 13 morphological traits, then a correlation heat map was made between the modules and the morphological traits (**Figure 6A**). For chemotype I, 17 of 20 up-regulated chemomarkers were included to the yellow metabolite module that closely positive correlated with leaf length (LL) in the heat map, and that implicated LL was one of the potential morphological markers of chemotype I. Analogously, for chemotype II, III, and IV, CRV, LBH and LT were their corresponded potential morphological markers, respectively, after examining the correlations between morphological traits and chemomarkers through the MEbrown module, the MEblue module, the MEgreen module, the MEpink module, and the MEyellow module (**Supplementary Table 13**).

Five flavonols (quercetin 3-(6″-acetylglucoside), quercetin-3-O-beta-glucopyranoside, quercetin-3-O-pentosidine, isoquercitrin, and kaempferol-3-O-glucoside) and three phloretic glycosides delphinidin, petunidin-3-O-glucoside, and cyanidin-3-O-alpha-arabinoside, were significantly negative correlated to LL after further correlation analysis between six key metabolite modules and eight morphological traits (**Figure 6B** and **Supplementary Table 14**). In addition, quercetin-3-O-pentosidine and isoquercitrin were of high importance degrees when their contents analyzed as dependent variables in RF regression analysis with LL, leaf area (LA), and leaf perimeter (LP), and that also suggested the three traits could be the morphological markers denoting the contents of the two metabolites in *E. ulmoides* leaves (**Figure 6C**).

## 4 Discussions

*E. ulmoides* has been considered of limited intraspecific variation as a monocotyledonous tree species of single family and single genus (Li et al., 2018a), and limited genetic diversity in the breeding are concerned by many *E. ulmoides* researchers. Accurate evaluation of the chemical composition and chemotype classification of germplasm resources could effectively expand the genetic base, promote the breeding potential and accelerate the directed breeding of *E. ulmoides.* Related studies had been conducted in diverse tea populations (Yu et al., 2020). Chemical components in different tissues of *E.ulmoides* including leaves, seeds and barks had been determined by ultra-high-performance liquid chromatography-tandem time-of-flight mass spectrometer (UHPLC-QTOF/MS) untargeted metabolomics, and finally 2,373 metabolites were identified in total (Chen et al., 2022). Besides, the dynamic metabolic models for leaf growth and development of *E.ulmoides* were constructed by integrated uses of widely targeted metabolomics and transcriptomics (Li et al., 2019). However, few studies employed metabolomics to wholly determine and evaluate the germplasm collections of *E.ulmoides*, expect of a recent study on the core collections of male flower (Liu et al., 2020). In present study, 1100 metabolites were determined by GC-MS and LC-MS/MS untargeted metabolomics in 579 samples, which sketched a comprehensive and explicit metabolite map of *E. ulmoides* leaves. The chemical variations in different collections were systematically displayed, and that provided holistic approaches and laid important foundations for germplasm resource evaluation, leaves quality control, metabolic regulation and directed breeding of *E. ulmoides*.

Recently, advanced machine learning algorithms including SOM and deep belief networks (DBN) were increasingly employed to deal with the high dimensional data in plant omics studies. As an unsupervised artificial neural network, SOM soft clustering was of high generalization ability and outperformed the K-means hard clustering in clustering similar characteristic data in regions of same network topology by self-organized learning space distribution of the eigenvectors (Crespo et al., 2020). RF was a compositive supervised learning method and could be regarded as an extension of the decision tree, which proceeded classification and regression of high dimensional data without dimension reduction and measured the relative importance of variables to the classification results, and that made it of clear advantages in processing large datasets (Chavent et al., 2021). OPLS-DA model was effective in examining the similarities or differences in specified groups, and performed internal and external validation to evaluate the effectiveness of the constructed model (Triba et al., 2015). The comprehensive use of the SOM, RF and OPLS-DA in chemotype classification of *E. ulmoides* leaves through analyzing matrices of complex sample size and metabolite quantity offered highly reliable and accurate results that were superior to K-means classification generally used in previous studies. However, for the classification principles, standards, and naming rules for specific chemotype have not been unified, the present classification of the leaf chemotype based on the content of metabolites just confirmed that chemical differentiations existed in the classified groups. The formulations of classification standards, naming rules and sub-divisions of the present classification should be given priority and issued in future research. Similar to the results from male flowers of *E. ulmoides* (Liu et al., 2020), collections belonged to the leaf chemotypes did not corresponded to their geographical distributed regions, indicating the variation in the leaf chemotypes might originate from intra-population variation and were broadly consistent with the genetic variations of *E. ulmoides* collections (Wuyun et al., 2018).

One or several metabolites can be used as biomarkers to identify collections belonged to specific chemotype, morphological type or genotype in the plant germplasm resource, and even to some biological processes, for instance, hybrid advantage of yield can be predicted by metabolites levels in the tyrosine metabolic pathway (Dan et al., 2021). 103 chemomarkers mostly belonged to terpenoids, flavonoids, phenolic glycosides, and lipids were screened on basis of values of VIP, p, and FC, and additionally, variable importance in RF model. Differences in the plant chemotypes might not be displayed in metabolites composition but also in morphological, growing and developmental traits. To chemotypes formation of *Tanacetum vulgare*, it was concluded that the number and diameter of flower heads, flowering period, and pollen nutritional quality were the significant indicators (Eilers et al., 2021). The morphological markers of the *E. ulmoides* leaf chemotypes were screened by hunting the correlations between the chemomarkers and morphological traits. The integrated application of chemomarkers and morphological markers will contribute to identify the chemotype of germplasm resources, and accelerate the efficiency in *E. ulmoides* breeding.

Correlation analysis between certain metabolites and extrinsic traits was important for understanding the molecular mechanisms of phenotypic variation (Chen et al., 2016). The genetic relationship between metabolism and phenotype in wheat had been revealed by combined analysis of metabolite-growth trait correlations and quantitative trait locus (Shi et al., 2020). Besides, the grain shape and stress resistance of rice was regulated by rice glycosyltransferase GSA1 modulating the phenanthrene metabolism (Dong et al., 2020). The predictability of metabolites values to LDN, CRV, LP, and SLDW of *E. ulmoides* reached to 0.5694, 0.5502, 0.4954, and 0.3914, respectively, by LASSO. This is comparable to the results from recombinant inbred line (RIL) in 145 wheat, with the predictability to grain per spike and plant height reaching to 0.51 and 0.46 by LASSO (Shi et al., 2020). In addition to significant negative correlations were observed between the content of isoquercitrin from target determination and the external traits such as LL, the contents of quercetin-3-O-pentosidine and isoquercitrin were closely correlated to LL, LA, and LP by RF regression analysis, and these results indicated quercetin derivatives might take an important role in the development of leaf shape of *E. ulmoides*.

## 5 Conclusions

A set of GC-MS and LC-MS/MS non-targeted metabolomics methods including sampling processing, metabolite extraction and determination, metabolite annotation and quantification, and data calibration was established for leaves of *E. ulmoides*, providing a basis for disclosing the metabolic diversity and variation, chemotype classification, and biomarkers screening for the germplasm resource. 1,100 metabolites belonged to 18 categories and contained 120 active ingredients for TCM and 85 disease-resistant metabolites were identified in leaves of 193 core collections of *E. ulmoides*. The integrated uses of unsupervised SOM, supervised OPLS-DA, and RF statistical methods were suitable to process classification and markers screening basing on the non-targeted metabolomics data. 103 chemomarkers corresponding to four established leaf chemotypes of *E. ulmoides* were screened, and the morphological markers linked to the leaf chemotypes were examined. Quercetin derivatives may influence the growth and development of the leaf shape of *E. ulmoides*, and that require further studies to confirm.

## Data availability statement

The data presented in the study were deposited in the Metabolights repository, accession number MTBLS5958.

## Author contributions

PL designed and supervised the experiments; YM and QD conducted the experiment, analyzed the data, and wrote the paper; HD revised the manuscript, QW, LW, and LD performed sample collection. All authors read and approved the final version of the manuscript.
