# Supplementary material for: Analysis of chemotypes and their markers in leaves of core collections of Eucommia ulmoides using metabolomics
Source: Front Plant Sci. 2023 Jan 9;13:1029907. doi: 10.3389/fpls.2022.1029907 (PMC9868706; doi:10.3389/fpls.2022.1029907)
Supplement: Supplementary file 1 [file DataSheet_1.pdf]

Supplementary Figure 1. HPLC chromatogram. **(A)** Mixed standards. **(B)** Samples. (Note: 1.Aucubin 2.Geniposide acid 3.Protocatechuic acid 4.Chlorogenic acid 5.Catechin 6.Asperuloside 7.Methyl gallate 8.Pinoresinol diglucoside 9.Genipin 10.Rutin 11.Isoquercitrin 12.Quercetin 13.Kaempferol)

Supplementary Figure 2. Plots of total ion current of one quality control sample by GC-MS determination and LC-MS/MS determination. **(A)** GC-MS. **(B)** ESI^+ mode of LC-MS/MS. **(C)** ESI^- mode of LC-MS/MS.

Supplementary Figure 3. RSDs distribution of characteristic ions in the samples and QC by RF calibration. **(A)** Characteristic ions of the samples determined by GC-MS. **(B)** Characteristic ions of QC determined by GC-MS. **(C)** Characteristic ions of ribitol determined by GC-MS before RF calibration. **(D)** Characteristic ions of ribitol determined by GC-MS after calibration. **(E)** Characteristic ions of the samples determined by the ESI^+ mode of LC-MS/MS. **(F)** Characteristic ions of QC determined by the ESI^+ mode of LC-MS/MS. **(G)** Characteristic ions of the samples determined by the ESI^- mode of LC-MS/MS. **(H)** Characteristic ions of QC determined by the ESI^- mode of LC-MS/MS.

Supplementary Figure 4. Boxplot of 13 metabolites among four classified chemotypes.
