# Supplementary figures and images for: Analysis of chemotypes and their markers in leaves of core collections of Eucommia ulmoides using metabolomics
Source: Front Plant Sci. 2023 Jan 9;13:1029907. doi: 10.3389/fpls.2022.1029907 (PMC9868706; doi:10.3389/fpls.2022.1029907)

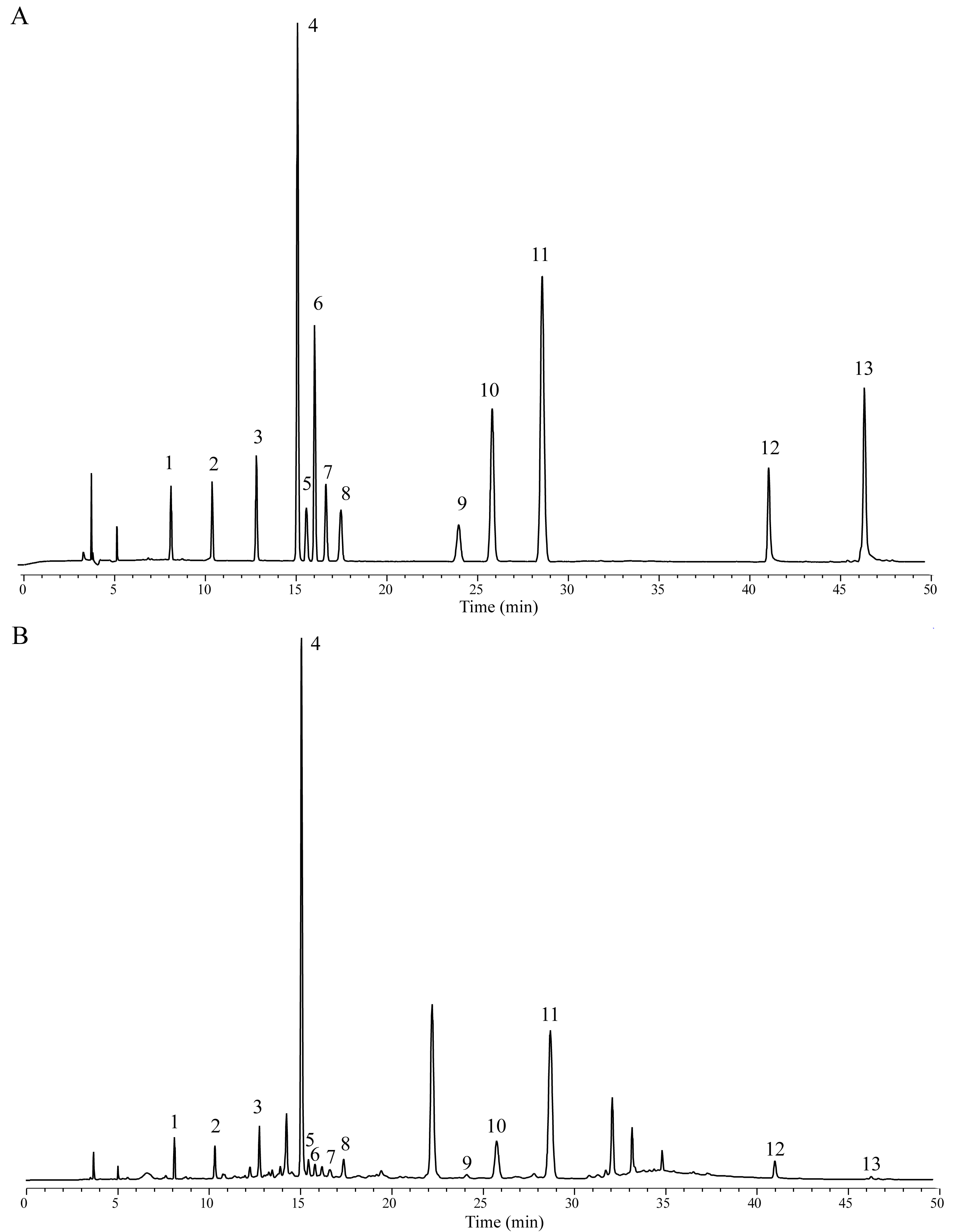

Supplement: Supplementary file 2 [file Image_1.tif]

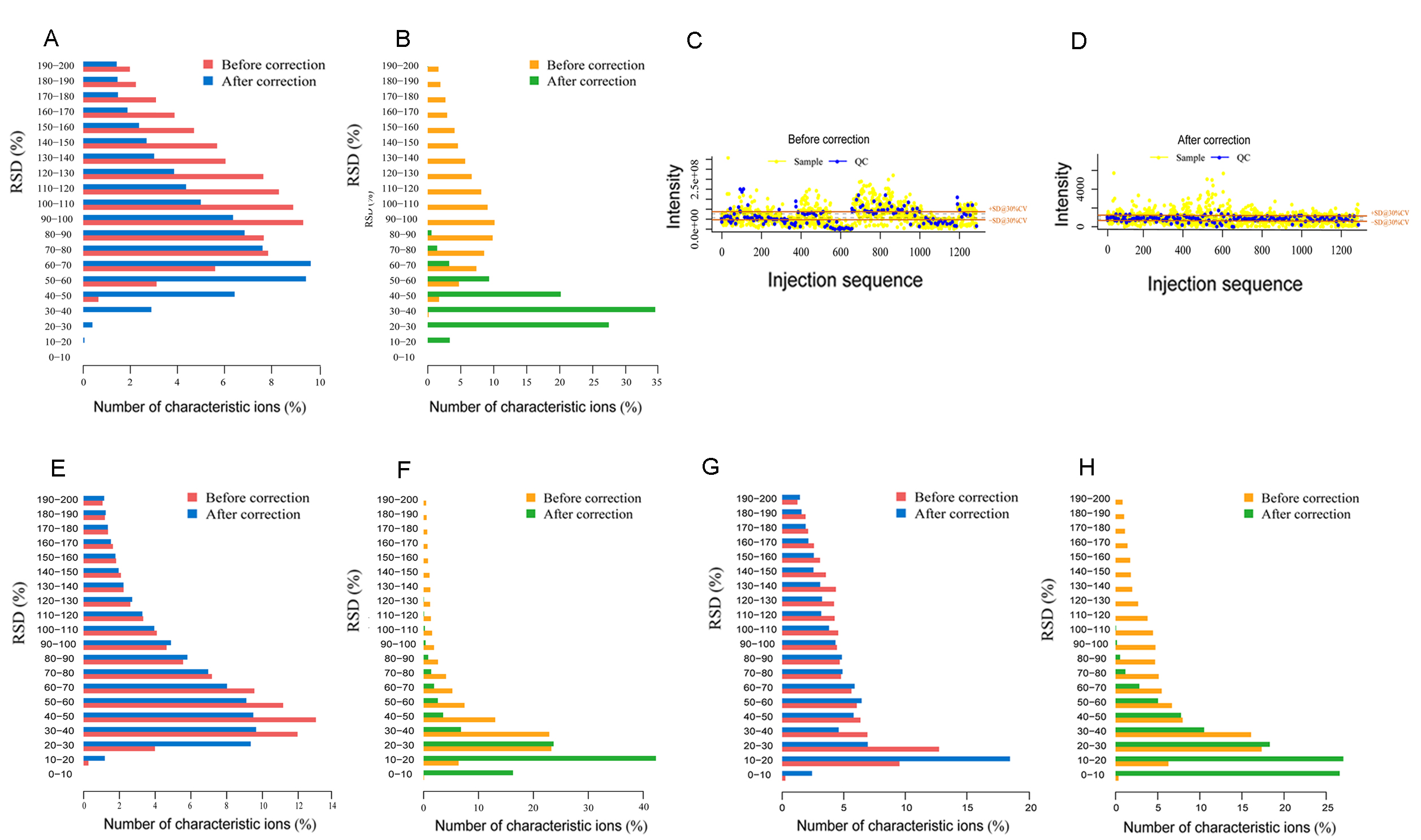

Supplement: Supplementary file 4 [file Image_3.tif]

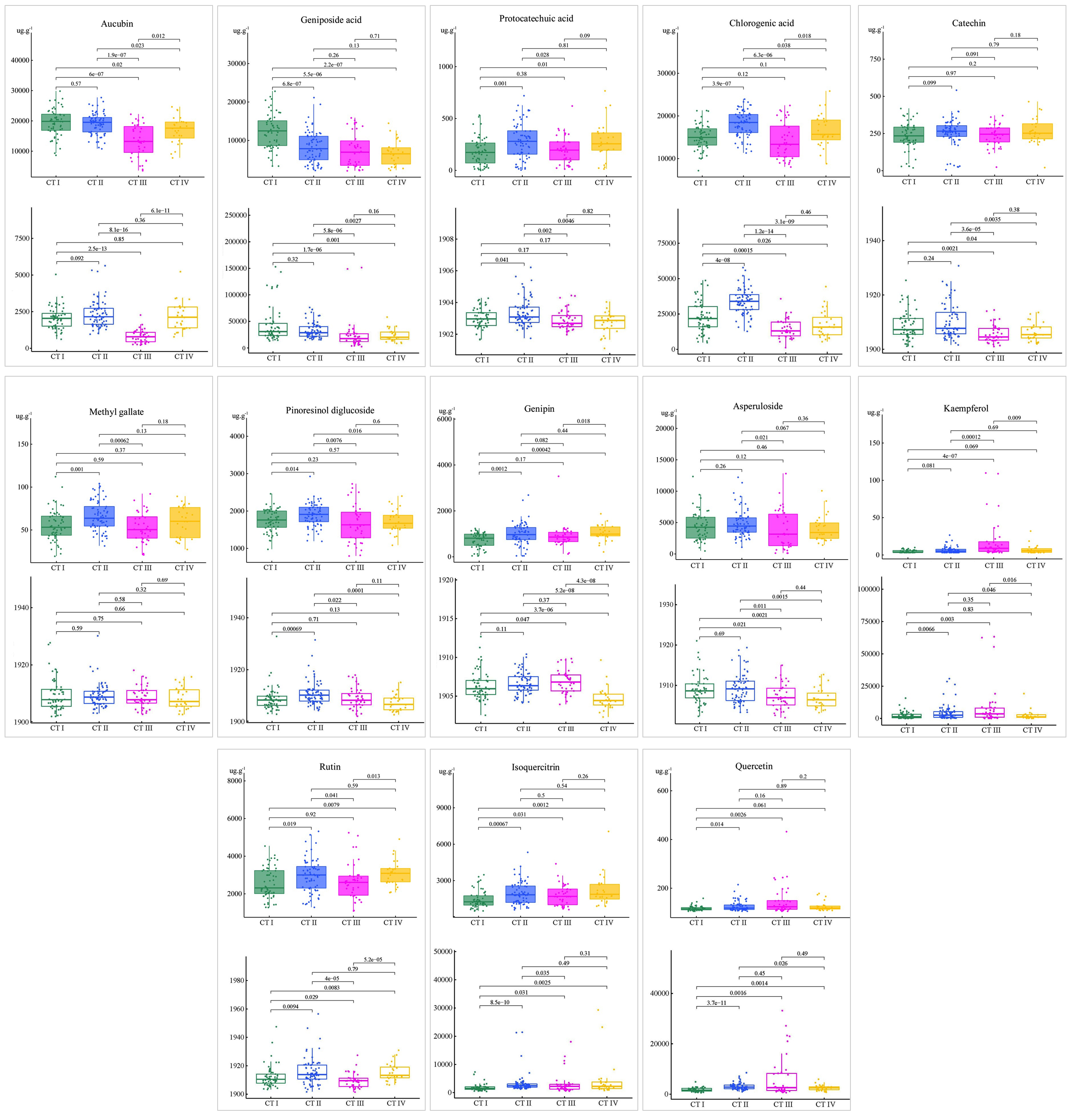

Supplement: Supplementary file 5 [file Image_4.tif]
